# Isoeugenin, a Novel Nitric Oxide Synthase Inhibitor Isolated from the Rhizomes of *Imperata cylindrica*

**DOI:** 10.3390/molecules201219767

**Published:** 2015-12-01

**Authors:** Hyo-Jin An, Agung Nugroho, Byong-Min Song, Hee-Juhn Park

**Affiliations:** 1 Department of Pharmacology, College of Korean Medicine, Sangji University, Wonju 220-702, Korea; hjan@sj.ac.kr; 2 Department of Agro-Industrial Technology, Faculty of Agriculture, Lambung Mangkurat University, Indonesia 70712, Indonesia; agung_tin@yahoo.com; 3 Department of Forest Science, College of Life Science and Natural Resources, Sangji University, Wonju 220-702, Korea; bmsong@sangji.ac.kr; 4 Department of Pharmaceutical Engineering, College of Health Science, Sangji University, Wonju 220-702, Korea

**Keywords:** *Imperata cylindrica*, isoeugenin, RAW264.7 macrophages, NO, HPLC

## Abstract

Phytochemical studies on the constituents of the rhizomes of *Imperata cylindrica* (*Gramineae*) were performed using high-performance liquid chromatography (HPLC). We also aimed to search for any biologically active substance capable of inhibiting nitric oxide (NO) formation in lipopolysaccharide (LPS)-activated macrophage 264.7 cells, by testing four compounds isolated from this plant. Four compounds, including a new chromone, isoeugenin, along with ferulic acid, *p*-coumaric acid, and caffeic acid were isolated and identified by NMR spectroscopy. The structure of isoeugenin was determined as 7-hydroxy-5-methoxy-2-methylchromone by the 2D-NMR technique. Among the four compounds, isoeugenin has the lowest IC_50_ value on the inhibition of NO production in LPS-activated macrophage RAW264.7 cells (IC_50_, 9.33 μg/mL). In addition, isoeugenin significantly suppressed the LPS-induced expressions of inducible nitric oxide synthase (iNOS), cyclooxygenase-2 (COX-2), and proinflammatory cytokines mRNA levels. Taken together, these results suggest that the anti-inflammatory activity of isoeugenin is associated with the down-regulation of iNOS, COX-2, and pro-inflammatory cytokines in RAW264.7 cells. Accordingly, our results suggest that the new chromone isoegenin should be considered a potential treatment for inflammatory disease.

## 1. Introduction

*Imperata cylindrica*, also called cogongrass, a traditional medicinal plant in Asia, is a widespread grass and is among the top 10 worst invasive weeds in the world. Traditionally, *I. cylindrica* is an Asian herb used to stop bleeding, as an expectorant, and as an anti-fever and anti-inflammatory agent. Recently, several compounds isolated from cogongrass exhibited medicinal properties, including anticancer properties, platelet aggregation inhibition, and hepatoprotective activities [1].

The major phytochemical constituents identified in *I. cylindrica* are phenylpropanoids [2], lignan glycosides [3], biphenyl ethers [4], sesquiterpenoids [5], phenolic compounds [6], lignans [5], and megastigmatrienone [7].

Several biologically active substances have been also reported to be present in *I. cylindrica*. These include the 5-lipoxygenase inhibitor cylindol A, vasodilator cylindrene, platelet aggregation inhibitor imperanene, and neuroprotectives 5-hydroxy-2(2-phenylethyl) chromone and 5-hydroxy-2(2-(2-hydroxyphenyl)ethyl) chromone. However, anti-inflammatory effect of *I. cylindrica* and its components have not been investigated.

Inflammation is a response to injury caused by physical or chemical noxious stimuli or microbiological toxins, and occurs in multiple pathologies, such as, arthritis, asthma, multiple sclerosis, inflammatory bowel diseases, and atherosclerosis [8]. In the inflammatory state, activated immune cells, such as macrophages secrete large amounts of proinflammatory cytokines and nitric oxide (NO). High levels of proinflammatory cytokines and NO in chronic inflammatory states can result in various pathological conditions [9]. In macrophages, lipopolysaccharide (LPS), a well-known endotoxin, induces the production of the inflammatory cytokines such as tumor necrosis factor-α (TNF-α), interleukin (IL)-6, and IL-1β, as well as inflammatory mediators, including NO and prostaglandin E_2_ (PGE_2_), that are synthesized by inducible NO synthase (iNOS) and cyclooxygenase-2 (COX-2), respectively [8,10].

Accordingly, control of the production of proinflammatory cytokines and NO in macrophages are current research topics for the development of new anti-inflammatory agents. As a part of our on-going screening project to evaluate the anti-inflammatory potentials of natural compounds, we isolated four compounds from the rhizomes of *I. cylindrica* and investigated their anti-inflammatory effects on LPS-stimulated RAW264.7 macrophages.

## 2. Results and Discussion

### 2.1. Isolation and Characterization of Compounds

The CHCl_3_ fraction fractionated from the MeOH extract of *I. cylindrica* was subjected to column chromatography to afford four compounds. The ^1^H-NMR spectrum of compound **1** measured in DMSO-*d*_6_ exhibited a vinylic methyl signal at δ 2.52. A singlet peak of H-3 at δ 5.60, the two doublet peaks of H-6 and 8 appearing as *meta*-coupled peaks (*J* = 1.8 Hz), together with a peak attributed to a OCH_3_ group at δ 3.93 (3H, s) suggests that compound **1** belongs to the chromone-type family of compounds.

The representative chromone-type compounds include eugenin, noreugenin, eugenitin, isoeugenitin, and isoeugenitol [11]. All of these have two CH_3_ groups, but some of them do not have a OCH_3_. The HR-ESI-MS showed that compound **1** exhibited an *m/z* of 206.0579 corresponding to a [C_11_H_10_O_4_]^+^ molecular ion. Although the molecular formula of compound **1** is the same as that of eugenin, its NMR data was different from other chromone-type compounds, therefore, compound **1** is different from known chromone-type compounds. In particular, the literature [11] indicates that eugenin possessing a 5-OH exhibits a peak of δ_C_ 182.7 (in CDCl_3_); therefore, it is suggested that compound **1** has a 5-OCH_3_ instead of a 5-OH according to its δ 169.9 value (in DMSO).

**Figure 1:**
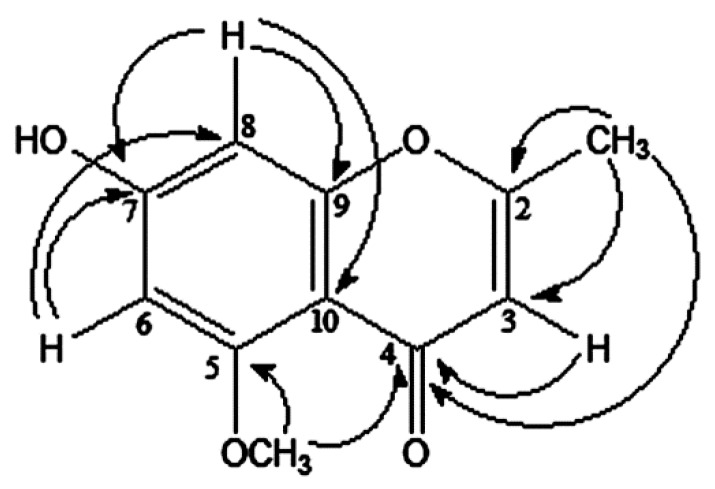
Structure of isoeugenin (**1**) isolated from *I. cylindrica* var. major and heteronuclear multiple-bond correlation spectroscopy (HMBC) correlation.

The peak at δ 2.52 (3H, s) is attributable to 2-CH_3_, commonly observed in this type of compounds [12] because the heteronuclear multiple-bond correlation spectroscopy (HMBC) spectrum showed its long-range coupling with δ_C_ 138.7, 87.2, and 169.9 as shown in Figure 1. A singlet peak (δ_H_ 5.60) attributable to H-3 overlapped with the peak at δ_C_ 169.9 of carbonyl. The position of 5-OCH_3_ assignable to δ_H_ 3.93 can be determined since the OCH_3_ peak is correlated with δ_C_ 169.9 (C-4). Therefore, the structure of compound **1** was determined as 7-hydroxy-5-methoxy-2-methyl-chromone, called isoeugenin. Compounds **2**–**4** were identified as ferulic acid [13], *p*-coumaric acid [14], and caffeic acid [15], respectively, after comparing our ^1^H- and ^13^C-NMR data with the literature data. Furthermore, Compounds **2**–**4** were identical to each corresponding standard compound on thin layer chromatography (TLC) and high performance liquid chromatography (HPLC).

**Figure 2:**
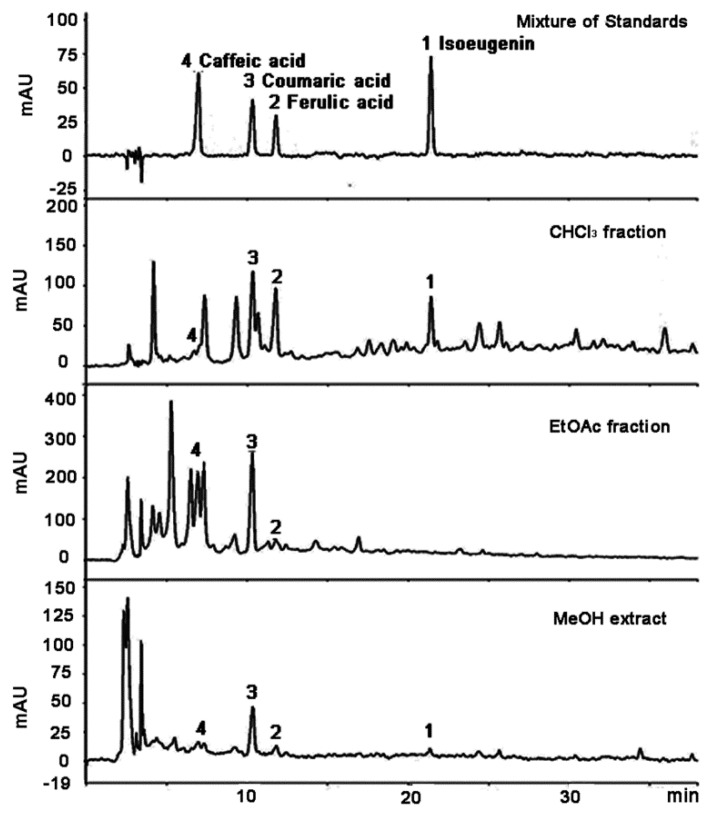
HPLC chromatograms of the MeOH extract of *I. cylindrica* and its fractions.

The four phenolic substances were analyzed using HPLC, and the chromatograms are shown in Figure 2. Regression equations were established as shown in Table 1, by estimating peak areas measured at six concentrations (12,5, 25, 50, 100, 200, and 1000 μg/mL). Each equation was verified for linearity at R^2^ > 0.999.

**Table 1:** Linearity of standard curves and detection/quantification limits for the standard compounds.

Standard Compounds	*t_R_* (min)	Calibration Equation (Linear Model) ^a^	Linear Range (μg/mL)	R^2 b^	LOD ^c^ (μg/mL)	LOQ ^d^ (μg/mL)
Caffeic acid	6.92	*y* = 747.05*x* + 83.33	12.50–200.0	0.9998	0.16	0.52
*p*-Coumaric acid	10.30	*y* = 245.73*x* + 95.83	12.50–200.0	0.9998	0.42	1.41
Ferulic acid	11.75	*y* = 276.37*x* + 41.66	12.50–200.0	0.9993	0.57	1.91
Isoeugenin	21.42	*y* = 40.468*x* + 30.26	25.00–1000.0	0.9998	4.19	13.97

^a^ *y*, peak area at 254 nm; *x*, concentration of the standard (μg/mL); ^b^ R^2^, correlation coefficient for 5 data points in the calibration curves (*n* = 4); ^c^ LOD, limit of detection (S/N = 3); ^d^ LOQ, limit of quantification (S/N = 10).

The contents of the four compounds were evaluated and revealed to be in the following order of abundance: isoeugenin (approximately 0.268 mg/g dry weight) > ferulic acid (approximately 0.042 mg/g) > *p*-coumaric acid (approximately 0.182 mg/g) > caffeic acid (approximately 0.020 mg/g). These four compounds were distributed mainly in the CHCl_3_ and EtOAc fractions as shown in Table 2. However, these substances are present in minute quantities in the hexane and BuOH fractions and isoeugenin was not detected in the EtOAc fraction.

**Table 2:** Content of analytes in the extract and fractions of *I. cylidrica*.

Analyte	MeOH Extract		Fractions (mg/g Extract)	
	(mg/g Dry Weight)	(mg/g Extract)	CHCl_3_	EtOAc
Caffeic acid	0.020	0.15	0.32	4.59
*p*-Coumaric acid	0.182	1.32	3.73	16.18
Ferulic acid	0.042	0.30	4.89	2.97
Isoeugenin	0.268	1.94	28.09	n.d.
Total	0.512	3.71	37.03	23.74

### 2.2. Inhibitory Effect of Compounds on LPS-Induced NO Production

When the cytotoxicity of the MeOH extract and its fractions were evaluated in macrophage 264.7 cells, only the CHCl_3_ fraction exhibited cytotoxicity at more than 100 μg/mL. The MeOH extract and other fractions displayed almost no cytotoxicity below 100 μg/mL concentration. When the cytotoxicity of the isolated compounds was evaluated in the cells, isoeugenin has a little cytotoxicity at 100 μg/mL and no cytotoxicity below 50 μg/mL. Ferulic acid, coumaric acid, and caffeic acid have no cytotoxicity at 12.5, 25, and 50 μg/mL (data not shown).

The inhibitory effects of the isolated compounds on NO formation in the LPS-activated macrophage 264.7 cells are shown in Table 3. In this experiment, concentrations less than those showing cytotoxicity were chosen for the nitrite assay. Isoeugenin considerably reduced NO formation in LPS-activated cells, although other compounds exhibited very low activities. The IC_50_ value of isoeugenin on the inhibition of NO formation was 9.33 μg/mL.

**Table 3:** Inhibitory effect of components of *I. cylidrica* on the LPS-induced NO production.

Group	Concentration	NO Production (μM)	Inhibition (%)
NOR	-	11.90 ± 0.65	-
LPS	-	77.18 ± 0.82 ^#^	-
l-NIL (μM)	20	36.12 ± 1.56 ***	53.21 ± 2.02
Isoeugenin (μg/mL)	12.5	32.96 ± 0.47 ***	57.29 ± 0.61
	25	10.36 ± 0.16 ***	86.58 ± 0.20
	50	6.12 ± 0.16 ***	92.07 ± 0.20
Ferulic Acid (μg/mL)	25	79.05 ± 3.08	−2.42 ± 3.99
	50	79.67 ± 3.16	−3.22 ± 4.09
	100	72.56 ± 0.71 **	5.99 ± 0.91
Coumaric acid (μg/mL)	25	77.89 ± 1.49	−0.92 ± 1.92
	50	77.00 ± 2.46	0.23 ± 3.19
	100	72.83 ± 1.67 **	5.64 ± 2.16
Caffeic acid (μg/mL)	25	76.38 ± 1.49	1.04 ± 1.06
	50	74.87 ± 0.67	2.99 ± 0.87
	100	66.60 ± 0.41 ***	13.70 ± 0.53

Cells were pretreated with different concentrations (12.5, 25, 50, 100 μg/mL) of samples for 1 h, then with LPS (1 μg/mL), and incubated for 24 h. Normal (NOR) values were obtained in the absence of LPS and samples. l-NIL was used as a positive control at a concentration of 20 μM. ^#^ *p* < 0.05 *vs.* the normal controls; ** *p* < 0.01; *** *p* < 0.001 *vs.* 1 μg/mL LPS-treated cells; the significances of differences between treated groups were determined using ANOVA and Dunnett’s *post hoc* test.

### 2.3. Inhibitory Effect of Isoeugenin on the LPS-Induced iNOS and COX-2 Expressions

**Figure 3:**
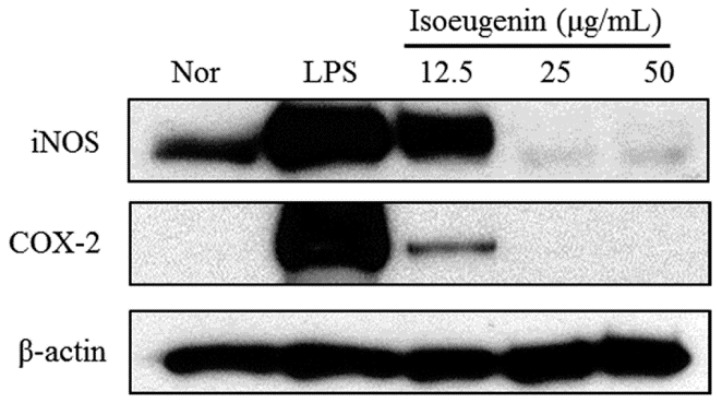
Inhibitory effects of isoeugenin on LPS-induced iNOS and COX-2 protein expressions in RAW264.7 cells. Cells were pretreated with different concentrations (12.5, 25, 50 μg/mL) of isoeugenin for 1 h, then with LPS (1 μg/mL), and incubated for 24 h. Total cellular proteins (30 μg) were resolved by SDS-PAGE, transferred to nitrocellulose membranes, and detected with specific antibodies, as described in the Experimental Section. NOR values were obtained in the absence of LPS and samples. LPS values were obtained in only LPS treatment. A representative immunoblot of three separate experiments is shown.

To determine whether the inhibitory effects of isoeugenin on NO productions are related to the modulation of iNOS and COX-2 enzymes, we examined their expression levels by western blotting. In unstimulated RAW264.7 cells, iNOS and COX-2 protein levels were undetectable. However, in response to LPS, the expression levels of iNOS and COX-2 were markedly upregulated, and isoeugenin significantly inhibited the LPS-stimulated iNOS and COX-2 expressions in a dose-dependent manner (Figure 3).

### 2.4. Inhibitory Effect of Isoeugenin on the LPS-Induced mRNA Levels of Cytokines

**Figure 4:**
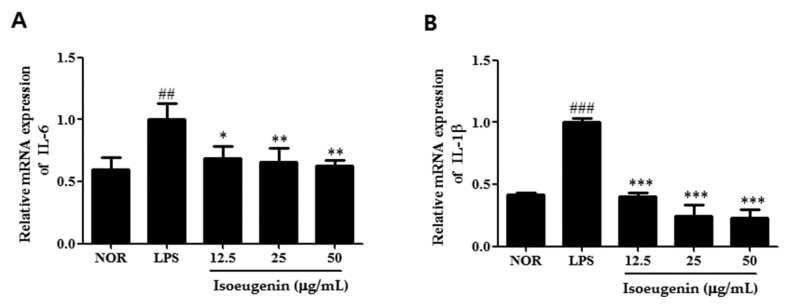

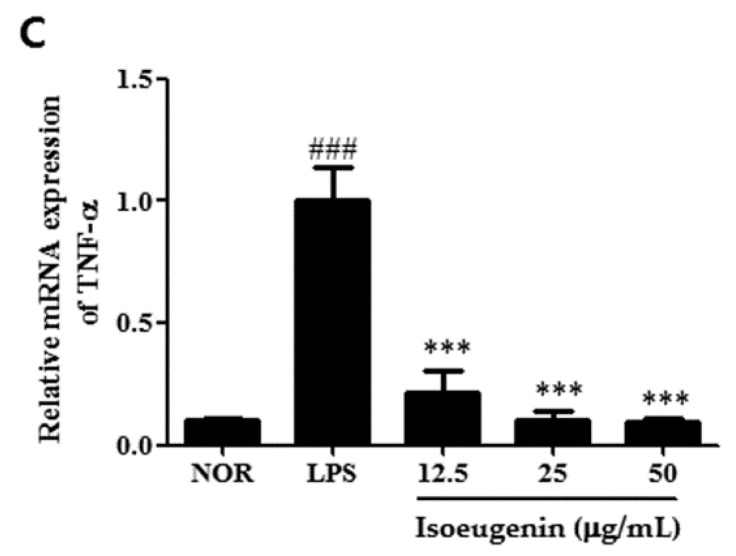
Inhibitory effect of isoeugenin on LPS-induced TNF-α (**A**); IL-6 (**B**); IL-1β (**C**) mRNA expression in RAW264.7 cells. Total RNA was prepared for the Real Time-PCR analysis of TNF-α, IL-6, IL-1β gene expression from RAW264.7 macrophage cells pretreated with different concentrations (12.5, 25, 50 μg/mL) of isoeugenin for 1 h followed by LPS (1 μg/mL) for 24 h. NOR values were obtained in the absence of LPS and samples. The experiment was repeated three times and similar results were obtained. Values represent means ± S.D. of three independent experiments. ^##^ *p* < 0.01, ^###^ *p* < 0.001 *vs.* the control group; * *p* < 0.05, ** *p* < 0.01, *** *p* < 0.001 *vs.* 1 μg/mL LPS-treated group; significant differences between treated groups were determined using ANOVA and Dunnett’s *post hoc* test.

To evaluate the effect of isoeugenin on the pro-inflammatory cytokines, we further examined the expressions of TNF-α, IL-6, and IL-1β in LPS-stimulated macrophages pretreated with isoeugenin by quantitative Real-time PCR. Pretreatment with isoeugenin was found to reduce LPS-induced TNF-α, IL-6, and IL-1β mRNA expressions in a concentration-dependent manner (Figure 4). These three cytokines are known to act as pro-inflammatory mediators *in vitro* and *in vivo*. TNF-α exhibits its pro-inflammatory activity by regulating several intercellular and vascular cell adhesion molecules, which results in the recruitment of leukocytes to sites of inflammation [16]. IL-6 is a cytokine released by LPS-activated monocytes and plays a crucial role in immune response [17]. For example, the overexpression of IL-6 is involved in pathological conditions, such as, rheumatoid arthritis [18]. IL-1β is normally produced in response to infection, injury, or immunologic challenge; at minimal concentrations, it causes fever, hypotension, and production of additional proinflammatory cytokines, such as IL-6 [19]. Collectively, isoeugenin has the potential for inhibition not only iNOS and COX-2 expression but also a wide range of the pro-inflammatory genes regulated.

## 3. Experimental Section

### 3.1. General Information

Chromatographic isolation was performed on the open columns using normal or reverse stationary phases. Spectra of the isolated compounds were measured using a NMR spectrometer (Bruker Co., Rheinstetten, Germany). A quantitative analysis was performed on the HPLC system (Varian Co., Palo Alto, CA, USA) consisting of two Prostar 210 pumps, a Prostar 325 UV-vis detector and a Shiseido Capcell PAK C18 column (5 μm, 4.6 mm × 250 mm, Japan). Dulbecco’s modified Eagle’s minimum essential medium (DMEM), fetal bovine serum (FBS), penicillin, and streptomycin were obtained from Life Technologies Inc. (Grand Island, NY, USA). iNOS, COX-2, and β-actin monoclonal antibodies and the peroxidase-conjugated secondary antibody were purchased from Santa Cruz Biotechnology, Inc. (Santa Cruz, CA, USA).

### 3.2. Plant Material

The rhizome of *I. cylindrica* was purchased from the Chun-Il medicinal herb store in Wonju City, Korea. This medicinal herb was identified by Prof. Byong-Min Song (Department of Forestry Science, Sangji University, Wonju City, Korea). The voucher specimen (natchem #68) was deposited in the laboratory of Natural Product Chemistry, Department of Pharmaceutical Engineering, Sangji University.

### 3.3. Extraction and Fractionation

The plant material (3 kg) consisting of the rhizome of *I. cylindrica* was extracted with MeOH (4 L) under reflux for 5 h and repeated three times. The extracted solution was filtered and evaporated under reduced pressure to give a MeOH extract (501.7 g). This extract was suspended in H_2_O and fractionated in a separating funnel with hexane (0.8 L) three times. The hexane-soluble portion was concentrated to dryness *in vacuo* to give a hexane fraction (24.40 g). Similarly, the remaining H_2_O layer was further successively fractionated with CHCl_3_, EtOAc, and BuOH, and then concentrated. The yields of the CHCl_3_, EtOAc, and BuOH fractions were 14.56 g, 12.58 g, and 136.1 g, respectively. The concentration of the final aqueous phase gave an H_2_O fraction (348.8 g).

### 3.4. Isolation of the Componds

The CHCl_3_ fraction (10 g) was chromatographed on a silica gel column (280 g, 5 cm × 35 cm) using CHCl_3_–MeOH-H_2_O (10:2:2, lower layer). Forty-five fractions (fr.4–fr.5; 60 mL (each)) were then grouped into 10 subfractions (Gr.1–Gr.10) according to their band profiles in TLC.

To obtain the main component from Gr.2, silica gel (55 g, 3 cm × 30 cm) column chromatography was performed using CHCl_3_–MeOH–H_2_O (10:2:2, lower layer) to yield compound **1**. Further, to obtain the main component from Gr.6, silica gel (60 g, 3 cm × 30 cm) column chromatography was undertaken using CHCl_3_–MeOH–H_2_O (10:2:1, lower layer) to yield compound **2**. To obtain the main component from Gr.8, it was chromatographed on silica gel (55 g, 3 cm × 30 cm) with the solvent CHCl_3_–MeOH–H_2_O (10:2.2:1, lower layer) to yield compound **4**.

Compounds **2**, **3**, and **4** were identified as ferulic acid, *p*-coumaric acid, and caffeic acid, respectively, by analysis of their corresponding ^1^H- and ^13^C-NMR spectra. The three compounds **2**–**4** were the same as standards of each compound by TLC and HPLC. The retention times of *p*-coumaric acid, ferulic acid and caffeic acid were 10.30 min, 11.75 min, and 6.92 min, respectively, on the HPLC chromatogram. On the other hand, compound **1** with a retention time of 21.42 min on the HPLC chromatogram was structurally determined to be isoeugenin through the interpretation of 2D-NMR spectra including ^1^H-^1^H correlation spectroscopy (COSY), ^1^H-^13^C COSY, HMBC, and nuclear Overhauser effect spectroscopy NMR spectra.

*7-Hydroxy-5-methoxy-2-methylchromone* (*isoeugenin*, **1**): Yellow gum; UV λ_max_ MeOH nm (log ε): 209 (4.22), 307 (3.87); ^1^H-NMR (600 MHz, dimethyl sulfoxide (DMSO-*d*_6_): 6.59 (1H, d, *J* = 1.8 Hz, H-8), 6.55 (1H, d, *J* = 1.8 Hz, H-6), 5.60 (1H, s, H-3), 3.93 (1H, s, OCH_3_), 2.52 (3H, s, 2-CH_3_); ^13^C-NMR (150 MHz, DMSO-*d*_6_): 138.7 (C-2), 87.2 (C-3), 169.9 (C-4), 162.2 (C-5), 101.0 (C-6), 160.9 (C-7), 116.5 (C-8), 156.6 (C-9), 106.6 (C-10), 57.0 (OCH_3_), 23.5 (CH_3_); High-resolution electrospray ionization mass spectrometry (HR-ESI-MS) *m*/*z* (rel. int.): 206.0605 (100, C_11_H_10_O_4_).

*Ferulic Acid* (**2**): Colorless gum; EI-MS (70 eV): *m*/*z* 194.1 (M^+^); ^1^H-NMR (500 MHz, CD_3_OD) and ^13^C-NMR (125.5 MHz, CD_3_OD) δ: [13].

*p-Coumaric Acid* (**3**): Colorless needles; m.p. 210–213 °C; EI-MS (70 eV): *m*/*z* 164.1 (M^+^), ^1^H-NMR (500 MHz, CD_3_OD) and ^13^C-NMR (125.5 MHz, CD_3_OD): [14].

*Caffeic Acid* (**4**): Yellow powder, m.p. 223–225 °C (dec.); EI-MS (70 eV): *m*/*z* 180.1 (M^+^), 163.06, 135.08; ^1^H-NMR (500 MHz, CD_3_OD) and ^13^C-NMR (125.5 MHz, CD_3_OD) [15].

### 3.5. HPLC Analysis

The HPLC system used for the present study consists of a Varian Prostar 210 solvent delivery module (Agilent Technologies, Santa Clara, CA, USA), Prostar 325 UV-Vis detector, and 20-μL sample loop. A Shiseido Capcell Pak C18 HPLC column (5 μm, 250 mm × 4.6 mm, Tokyo, Japan) was used. The two solvents used for gradient elution were solvent A 0.5% HOAc aqueous solution, and solvent B 0.5% HOAc-MeOH solution. Regression equations were established as shown in Table 1, by estimating peak areas measured at six concentrations (12, 5, 25, 50, 100, 200, and 1000 μg/mL). Each equation was verified for linearity at *R*^2^ > 0.999. Detection was performed at the fixed wavelength of 254 nm.

### 3.6. Preparation of Standard and Test Solutions

The four compounds, isoeugenin, ferulic acid, *p*-coumaric acid, and caffeic acid, which were isolated from *I. cylindrica* were used for preparation of standard solutions. Stock solutions (each 1000 μg/mL) that were made by dissolving each standard compound in MeOH were further diluted to produce working standard solutions. The rhizome of *I. cylindrica* was pulverized, added to 20 mL MeOH, and then extracted at 60 °C for 6 h using an ultrasonicator. The extracted solution was filtered through a syringe filter and used for sample solution. The other four sample solutions (each 1000 μg/mL) were prepared by dissolving hexane CHCl_3_, EtOAc, and BuOH fractions in MeOH.

### 3.7. Cell Culture and Sample Treatment

The RAW264.7 murine macrophage cell line was obtained from the Korea Cell Line Bank (Seoul, Korea). These cells were grown at 37 °C in Dulbecco’s modified Eagle’s medium containing 10% fetal bovine serum, 100 U/mL penicillin, and 100 μg/mL streptomycin in a humidified atmosphere of 5% CO_2_. Cells were incubated with various concentrations of the MeOH extract, fractions, or with positive controls (l-N6-(1-iminoethyl)lysine (NIL)), and then stimulated with LPS (1 mg/mL) for the indicated time. Various concentrations of the MeOH extract, four fractions, and isolated compounds dissolved in DMSO were added to the medium.

### 3.8. Measurement of Cell Viability by 3-(4,5-Dimethylthiazol-2-yl)-2,5-diphenyltetrazolium Bromide (MTT) Assay

Cells were incubated after being treated with various concentrations of four compounds for 24 h. This medium was further incubated for 4 h after adding 5 mg/mL MTT solution. After discarding the medium, the formazan crystals formed was dissolved in DMSO and then the absorbance measured at 570 nm using an Epoch microplate spectrometer (Biotek, Winooski, VT, USA).

### 3.9. Measurement of Nitrite in Culture Media

The concentration of NO produced in RAW264.7 cells was determined by measuring nitrite (NO_2_^−^) levels in the medium using Griess reagent (1% sulfanilamide in 5% phosphoric acid, 1% α-naphthylamide in H_2_O) as previous study [20]. After incubating the medium with the supernatant (50 μL) and Griess reagent (50 μL) for 15 min, the absorbance was measured at 540 nm with a microplate reader.

### 3.10. Western Blot Analysis

The cells were re-suspended in a commercial lysis buffer (PRO-PREP™, Intron Biotechnology, Seoul, Korea) and incubated for 20 min at 4 °C. Cell debris was removed by micro-centrifugation, followed by quick freezing of the supernatants. The protein concentration was determined using the Bio-Rad protein assay reagent according to the manufacturer’s instructions (Bio-Rad, Hercules, CA, USA). Aliquots of each protein sample (30 μg) were separated on a sodium dodecyl sulfate (SDS) polyacrylamide gel and transferred onto a polyvinylidene fluoride (PVDF) membrane. Membranes were incubated for 1 h with 5% skim milk at room temperature, followed by incubation overnight with a primary antibody (iNOS, COX-2 (Santa Cruz Biotechnology)) at 4 °C. Blots were washed three times with Tween 20/Tris-buffered saline (T/TBS) and incubated with a 1:1000 dilution of horseradish peroxidase-conjugated secondary antibody (Jackson Immunoresearch, Inc., Baltmore, MD, USA) for 2 h at room temperature. Blots were again washed three times with T/TBS, and then developed by enhanced chemiluminescence (GE Healthcare, Milwaukee, WI, USA).

### 3.11. Quantitative Real-Time PCR Analysis

Total RNA was isolated from the cells or liver tissue using a Trizol reagent (Invitrogen, Carlsbad, CA, USA). cDNA was obtained using isolated total RNA (2 μg), d(T)16 primer and AMV reverse transcriptase. Relative gene expression was quantified by use quantitative real-time PCR (Real Time PCR System 7500, Applied Biosystems, Foster, CA, USA) with SYBR Primix Ex Taq. The The oligonucleotide primers used in this study are listed below and were purchased from Bioneer (Seoul, Korea): for TNF-α were ATGAGCACAGAAAGCATGAT (forward) and TACAGGCTTGTCACTCG AAT (reverse); for IL-6 were TTCCATCCAGTTGCCTTCTTG (forward) and GGGAGTGGTATCC TCTGTGAAGTC (reverse); for IL-1β were GATCCACACTCTCCAGCTGCA (forward) and CAAC CAACAAGTGATATTCTCCATG (reverse); for GAPDH GACGGCCGCATCTTCTTGT(forward) and CACACCGACCTTCACCATTTT (reverse:), and the suitable size of synthesized cDNA was 200 bp. The results are expressed as the ratio of optimal density to GAPDH.

## 4. Conclusions

In the present study, we obtained four compounds from a MeOH extract of *I. cylindrica* rhizomes and tested them for anti-inflammatory activity. As part of our study, we identified the novel compound isoeugenin for the first time and also we demonstrated the anti-inflammatory effects of isoeugenin on LPS-activated RAW264.7 macrophages. Based on these findings, isoeugenin could be a useful pharmacologic tool for improving our understanding of basic cellular functions and suggest that isoeugenin be considered for evaluation as a potential treatment option for inflammatory diseases.
